# Comparative evaluation of saliva and nasopharyngeal swab for *SARS-CoV-2* detection using RT-qPCR among COVID-19 suspected patients at Jigjiga, Eastern Ethiopia

**DOI:** 10.1371/journal.pone.0282976

**Published:** 2023-03-13

**Authors:** Bawlah Tahir, Fitsum Weldegebreal, Firayad Ayele, Desalegn Admassu Ayana

**Affiliations:** 1 Department of Medical Laboratory Sciences, Jigjiga University, Jigjiga, Ethiopia; 2 School of Medical Laboratory Sciences, College of Health and Medical Sciences, Haramaya University, Harar, Ethiopia; University of Helsinki: Helsingin Yliopisto, FINLAND

## Abstract

**Background:**

Nasopharyngeal swab (NPS) remains the recommended sample type for Severe Acute Respiratory Syndrome Coronavirus-2 (SARS-CoV-2) diagnosis. However, the collection procedure causes discomfort and irritation to the patients, lowering the quality of the sample and exposing healthcare workers to risk. Furthermore, there is also a shortage of flocked swabs and personnel protective equipment in low-income settings. Therefore, this necessitates an alternative diagnostic specimen. The purpose of this study was to evaluate the performance of saliva against NPS for SARS-CoV-2 detection using RT-qPCR among COVID-19 suspected patients at Jigjiga, Eastern Ethiopia.

**Methods:**

Comparative cross-sectional study was conducted from June 28 to July 30, 2022. A total of 227 paired saliva and NPS samples were collected from 227 COVID-19 suspected patients. Saliva and NPS samples were collected and transported to the Somali Regional Molecular Laboratory. Extraction was conducted using DaAn kit (DaAn Gene Co., Ltd China). Veri-Q RT-qPCR was used for amplification and detection (Mico BioMed Co, Ltd, Republic of Korea). The data were entered into Epi-data version 4.6 and analyzed using SPSS 25. McNemar’s test was used to compare the detection rate. Agreement between NPS and saliva was performed using Cohen’s Kappa. The mean and median of cycle threshold values were compared using paired t-tests and the correlation between cycle threshold values was measured using Pearson correlation coefficient. P value < 0.05 was considered statistically significant.

**Results:**

The overall positivity rate of SARS-CoV-2 RNA was 22.5% (95% CI 17–28%). Saliva showed higher sensitivity (83.8%, 95% CI, 73–94.5%) than NPS (68.9%, 95% CI 60.8–76.8%). The specificity of saliva was 92.6% (95% CI, 80.6% - 100%) compared to NPS (96.7%, 95% CI, 87% - 100%). The positive, negative, and overall percent agreement between NPS and saliva was 83.8%, 92.6%, and 91.2% respectively (κ = 0.703, 95% CI 0.58–0.825, *P* = 0.00). The concordance rate between the two samples was 60.8%. NPS showed a higher viral load than saliva. There was low positive correlation between the cycle threshold values of the two samples (r = 0.41, 95% CI -1.69 to -0.98, *P* >0.05).

**Conclusion:**

Saliva showed a higher detection rate for SARS-CoV-2 molecular diagnosis than NPS and there was significant agreement between the two specimens. Therefore, saliva could be suitable and easily obtainable alternative diagnostic specimen for SARS-CoV-2 molecular diagnosis.

## Introduction

A novel Coronavirus named Severe Acute Respiratory Syndrome Coronavirus-2 (SARS-CoV-2) emerged in Wuhan, China, in late 2019 and caused Coronavirus disease- 2019 (COVID-19) [1]. Since then, it spread rapidly across the globe resulting global health threats [2, 3]. On March 11^th^, 2020, WHO declared COVID-19 as a global pandemic [4].

Globally over 600 million COVID-19 cases and over 6.47 million deaths have been reported as of September 02, 2022, WHO reports. In Africa, the total number of cases is over 9.2 million cases and over 493 thousand cases with 7,572 deaths in Ethiopia as of September 02, 2022 [5]. SARS-CoV-2 laboratory diagnosis plays a cornerstone role in COVID-19 management, control and prevention.

As per the Infectious Disease Society of America (IDSA), SARS-CoV-2 can be diagnosed by Real-Time quantitative Polymerase Chain Reaction (RT-qPCR) using specimens such as NPS, Oropharyngeal swab (OPS), mid-turbinate swabs and nasal swabs. Nevertheless, the NPS remains the gold standard specimen [6]. However, collecting this specimen requires trained health care worker involvement which in turn, poses a biosafety risk to the health care worker; it also causes discomfort and sometimes bleeding in the tissue of the patients limiting repeat tests [7]. In addition, due to the abundant shortage of the viral transport medium, flocked swabs, and personal protective equipment in low-income countries including Ethiopia, NPS collection is not possible during large-scale testing [8]. Thus, this diminishes the capacity of diagnostic testing and mass screening. Besides, the NPS collection technique could not be applied to all populations; particularly children and patients with contra-indications [9]. False negative and inconsistent results had also been related to NPS; which might be due to its technical complexity [10]. Furthermore, an incorrect NPS collection procedure could affect the quality of the sample and give rise to an indeterminate or inconclusive result [11]. Besides, in the study area, the population usually hesitates to be tested using a NPS due to its complexity in the collection and this necessitates the search for an alternative specimen. Saliva has recently been reported as an alternative choice for SARS-CoV-2 detection with a pooled sensitivity of 83.2% and specificity of 99.2% compared with pooled sensitivity and specificity of the NPS 84.8% and 98.9% [12]. However, there are limited data in Ethiopia. Therefore, this study is aimed at evaluating the performance of saliva specimen for SARS-CoV-2 molecular detection compared to standard NPS.

## Materials and methods

### Study area, design, and period

A comparative cross-sectional study design was conducted at Jigjiga University Sheik Hassan Yabere Referral Hospital (JJU- SHYRH) from June 28 up to July 30, 2022, to compare saliva sensitivity and specificity against NPS for SARS-CoV-2 detection using RT-qPCR among COVID-19 suspected patients. Jigjiga city is 630 km away from Addis Ababa. JJU- SHYRH gives milestone healthcare services to all Somali regional zones.

### Population, inclusion and exclusion criteria

The source population was all COVID-19 suspected patients in Jigjiga city. The study population was all COVID-19 suspected patients who had obtained healthcare services at JJU-SHYRH during the study. All COVID-19 suspected patients attending JJU-SHYRH were included. Patients who were under respiratory aid, suspects who were unwilling to participate, seriously sick patients & unable to communicate were excluded.

#### Sample size and sampling technique

The sample size was calculated using a single population proportion formula based on the assumption of 5% expected margins of error, considering 95% confidence interval, 5% non-response rate and by taking the prevalence of 17% [13]. The final sample size was 227; a paired 227 saliva and NPS samples. A convenience sampling technique was used to include voluntary COVID-19 suspected patients in JJU-SHYRH.

### Data collection methods

COVID-19 suspected patients over the age of 18 who provided informed consent with a signature were included. Structured questionnaires were used to collect information about sociodemographic characteristics. For children and participants under the age of 18, informed consent signed by their parents/guardians or relatives was obtained and necessary information was gathered. Data collectors were medical laboratory professionals who were trained on the study purpose as well as biosafety issues related to sample collection. They were also taught about proper specimen handling, integrity maintenance and management. The principal investigator oversaw the data collectors.

#### Sample collection, handling and transportation

The procedure for collecting saliva and the distinction between saliva and sputum samples were explained to study participants. COVID-19 suspected individuals were asked to collect around 1ml of random deep-throat saliva which was spitted into a sterile sputum collection container [14] under the supervision of a healthcare worker. The sample was transported in accordance with WHO interim guidance on COVID-19 specimen collection and transportation [15]. One milliliter of viral transport media (VTM) was added as soon as the samples arrived in the Molecular-biology Laboratory [16].

The collection of the saliva was not restricted to timing and eating for simplifying the collection. NPS were collected using sterile flocked swabs and inserted into tubes containing viral transport medium as per the standard protocol by a trained health professional [17]. As per the standard protocol, upon arrival, all clinical specimens in the package were disinfected on the outer and inner sides of the ice box or triple package using 70% alcohol [18]. Each specimen was checked for completeness, registration and for being transported in a triple package with ice to the Somali Regional laboratory COVID-19 molecular center. All the specimens arrived and were processed as soon as possible within 4 hours. However, if the delay was not avoidable, specimens were stored at 2–8°C for 72 hours or -20°C for ≥ five days [19].

### Laboratory analysis

#### Nucleic acid extraction and master-mix

Total nucleic acid extraction was conducted using the spin column method with DaAn extraction kit (DaAn Gene Co., Ltd China). In summary, 200μl from the NPS and 200μl of saliva were taken into a separate 2ml eppendorf micro-centrifuge tube. 200μl of lysing working solution was added and subsequently, the mixture was heated at 72°c for 10 minutes which in turn, enhances cell breakage and deactivation of the virus. Several washing steps were carried out and lastly 50 μl of preheated eluent was added to the washed eppendorf tube. Then, pure RNA was obtained.

Veri-Q nCoV-QM detection kit (Mico BioMed Co., Ltd., Republic of Korea) was used whereby 5μl of master mix, 1μl of probe/primer mixture (MM) and 1μl of internal positive control (IPC) were mixed into 1.5ml micro-centrifuge tube per each sample and vortexed for few seconds. The 7μl of the mixture was transferred into 1.5 μl of eppendorf tube. Lastly, transported into the template addition room.

### Template addition, amplification and result interpretation

Extracted and eluted RNA was taken into the template addition room and 3μl of the RNA was added into the mixture, vortexed thoroughly. 8.2 μl of the solution was finally transferred into a lab chip and tested by using one-step Veri-Q RT-PCR 316.

Veri-Q nCoV-QM detection kit (Mico BioMed Co., Ltd., Republic of Korea) targets two main regions of the SARS-CoV-2 and one for human RNA with specific fluorescent reporters; these are ORF3a region which is reported by FAM, N-gene reported by Cy5 and Internal positive control (IPC) which is reported by Texas-red [20]. Samples were classified as positive for SARS-CoV-2 when ORF3a, N-gene and IPC gene are detected and when only ORF3a and IPC are detected at cycle threshold (Ct)value <40. In addition; when only the N-gene together with IPC is amplified, the test was repeated. Negative when only IPC gene is amplified [20]. The viral load distribution between saliva and NPS was assessed based on the Ct-value [21].

RT- qPCR results of saliva and NPS were interpreted by two different laboratory professionals and finally reviewed by the most experienced personnel. The samples with indeterminate results were processed again and interpreted according to kit’s instruction. Four different controls such as non-template control, internal positive control, and known negative and positive control were used to maintain the quality of the result.

### Study variables

#### Dependent variables

Evaluating saliva sensitivity against nasopharyngeal swab

### Statistical analysis

The laboratory result was registered on the questionary, entered into Epi data version 4.6 and analyzed in Statistical Package for Social Sciences (SPSS) version 25. Data were analyzed for normality and descriptive statistics were presented as a number and percentage (%) for categorical variables and mean ± standard deviation (SD) or median (range) for continuous variables by using SPSS version 25. McNemar’s test was used to compare the detection rate for the two sampling methods in terms of the number of positive patients. Agreement between NPS and saliva was performed using Cohen’s Kappa (κ) statistics. Positive, negative and overall percent agreement of the paired saliva and NPS were calculated. Ct values were compared using paired *t-*test. Correlation between Ct values of NPS and saliva was assessed using Pearson correlation coefficient. A *P* value < 0.05 was considered statistically significant.

### Ethical consideration

Ethical clearance was obtained from Haramaya University, College of Health and Medical science, Institutional Health Research Ethics Review Committee (IHRERC) (Ref. No. IHRERC/122/2022). Official letter of support was written to JJU-SHYRH Hospital administration. The study subjects were informed about the procedures and significance of the study. Any information about the data was kept confidential and the result was only communicated to an authorized concerned body. Every study participant had the right to refuse to take part in the study and those with no willingness were not forced to be included in the study. The possible COVID-19 transmission was prevented by wearing a facemask and keeping a distance of at least 2 meters among the participants and data collectors worn personnel protective equipment.

## Result

### Positivity rate of SARS-CoV-2 in NPS and saliva samples

Two-hundred twenty-seven paired saliva and NPS samples were collected from 227 COVID-19 suspected patients. The majority 64.3% (146/227) of the study participants were male. The mean age of the study participants was 36.33 (SD±14.19) and the minimum and maximum age were 1–79 years respectively. About 72.7% (165/227) of the participants were outpatients. In addition, most 79.7% (181/227) of the study participants were urban residents (Table 1).

**Table 1:** Demographic characteristics of study participants for comparative evaluation of saliva and NPS for SARS-CoV-2 detection using RT-qPCR among COVID-19 suspected patients at Jigjiga, Eastern Ethiopia.

Variable	Category	Frequency	Percentage	COVID-19	
				Positive N (%)	Negative N (%)
Sex	Male	146	64.3%	35 (15.4%)	111(48.9%)
	Female	81	35.7%	16 (7.1%)	65 (28.6%)
Age	0–14	13	5.7%	5 (2.2%)	8 (3.5%)
	15–24	22	9.7%	5 (2.2%)	17 (7.5%)
	25–54	161	70.9%	28 (12.3%)	133 (58.6%)
	55–64	22	9.7%	10 (4.4%)	12 (5.3%)
	≥65	9	4.0%	3 (1.3%)	6 (2.6%)
Residence	Urban	181	79.7%	43 (23.8%)	138 (76.2%)
	Rural	46	20.3%	8 (17.4%)	38 (82.6%)
Patient type	Outpatient	165	72.7%	29 (12.8%)	136 (59.9%)
	Inpatient	62	27.3%	22 (9.6%)	40 (17.62%)

SARS-CoV-2 RNA positivity rate in male (15.4%, 35/227) participants was higher than female (7.1%, 16/227). An among positive patients of both sexes; individuals aged between 25–54 years old were mostly (12.3%, 161/227) affected (Table 1). An among positive male participants, patients aged between 25–54 years had the highest positivity rate(10.96%, 16/146) followed by 55–64 years age group (5.5%, 8/146) (Table 2).

**Table 2:** SARS-CoV-2 RNA positivity rate difference sex with their respective age groups for comparative evaluation of saliva and NPS for SARS-CoV-2 detection using RT-qPCR among COVID-19 suspected patients at Jigjiga, Eastern Ethiopia.

Sex with age category			Frequency	Percentage	COVID-19 status	
					Positive	Negative
Male	Age group	0–14	11	7.5%	5 (3.4%)	6 (4.1%
		15–24	5	3.4%	3 (2.1%%)	2 (1.4%)
		25–54	108	73.97%	16 (10.96%)	92 (63%)
		55–64	14	9.6%	8 (5.5%)	6 (4.1%)
		≥65	8	5.5%	3 (2.1%)	5 (3.4%)
	**Total**		146	100%	35 (24%)	111 (76%)
Female	Age group	0–14	2	2.5%	0	2 (2.5%)
		15–24	17	20.98%	2 (2.5%)	15 (18.5%)
		25–54	53	65.4%	12 (14.8%)	41 (50.6%)
		55–64	8	9.9%	2 (2.5%)	6 (7.4%)
		≥65	1	1.2%	0	1 (1.23%)
	**Total**		81	100%	16 (19.8%)	65 (80.2%)

The overall positivity rate of SARS-CoV-2 in both saliva and NPS in this study was 22.5% (51/227), (95% CI 17–28%). The separate positivity rate of SARS-CoV-2 in saliva and NPS was 19.8% (45/227), (95% CI; 14.5%– 25%) and 16.3% (37/227), (95% CI; 11.5%– 21.1%) respectively (Table 3).

**Table 3:** Positivity rate of SARS-CoV-2 in saliva and NPS for comparative evaluation of saliva and NPS for SARS-CoV-2 detection using RT-PCR among COVID-19 Suspected patients at Eastern Ethiopia (n = 227).

RT-PCR result	Saliva	Nasopharyngeal swab (NPS)
	N (%)	N (%)
Positive	45 (19.8%)	37(16.3%)
Negative	182 (80.2%)	190 (83.7%)
Total	227 (100%)	227 (100%)

Thirty-one patients out of 51 positive participants (60.8%, 31/51) had concordant results, meaning SARS-CoV-2 was found in both NPS and saliva. Twenty patients (39.2%,20/51) had discordant results where SARS-CoV-2 was either detected in saliva or NPS. An among patients with the discordant result, 27.5% (14/51) of the patients had the virus detected in saliva but not in NPS, and 11.8% (6/51) of the patients had the virus detected in NPS but not in saliva (Table 4).

**Table 4:** Concordant and discordant rate between the saliva and NPS (only positives) for comparative evaluation of saliva and NPS for SARS-CoV-2 detection using RT-PCR among COVID-19 suspected patients at Eastern (n = 51).

Samples	Positive (N %)	Concordant rate (%)	Discordant rate (%)
Both in saliva and NPS	31 (60.8%)	60.8 (31/51)	39.2% (20/51)
Only in Saliva	14 (27.4%)		
Only in NPS	6 (11.7%)		
Total	51 (100%)		

In NPS, the majority 52.9% (27/37) of the positive samples had gotten amplification in both target genes (ORF3a and N) followed by amplification only in ORF3a target gene (13.7%, 7/37). Moreover; in saliva, the majority 60.8% (31/45) of the positive saliva samples had amplification in both SARS-CoV-2 target genes (ORF3a and N) followed by amplification in only ORF3a (19.6%, 10/45). Only 7.8% (4/45), of positive saliva samples, got only N-gene amplification (Table 5).

**Table 5:** Positive NPS and saliva samples when both ORF3a and N genes amplified vs when only N gene amplified for comparative evaluation of saliva and NPS for SARS-CoV-2 detection using RT-PCR among COVID-19 suspected patients at Jigjiga, Eastern (n = 51).

**NPS–Positive samples**		
**Genes- amplified**	**Frequency**	**Percentage**
Both ORF3a and N	27	52.9%
Only ORF3a	7	13.7%
Only N	3	5.9%
**Total**	**37**	**72.5%**
**Saliva-Positive samples**		
Both ORF3a and N	31	60.8%
Only ORF3a	10	19.6%
Only N	4	7.8%
**Total**	**45**	**88.2%**

### Sensitivity and specificity of saliva against NPS for SARS-CoV-2 detection

The sensitivity of saliva was 83.8% (95% CI, 73–94.5%) whereas the NPS sensitivity was 68.8% (95% CI 60.8–76.8%). The specificity of saliva was 92.6% (95% CI, 80.6% - 100%) whereas NPS specificity was 96.7% (95% CI, 87% - 100%). There was a higher detection rate in saliva than in NPS though the difference was not statistically significant (P = 0.12) (Table 6). Saliva showed considerable specificity for SARS-CoV-2 compared to NPS as confidence interval overlaps.

**Table 6:** Comparison of saliva sensitivity against NPS and agreement between saliva and NPS swab for comparative evaluation of saliva and NPS for SARS-CoV-2 detection using RT-qPCR among COVID-19 suspected patients at Jigjiga, Eastern Ethiopia (n = 227).

		NPS		Sensitivity		Specificity		*P*
		+ve	-ve	Saliva	NPS	Saliva	NPS	
				(95%, CI)	(95%, CI)	(95%, CI)	(95%, CI)	
Saliva	+ve	31	14	83.8%	68.8%	92.6%	96.7%	0.12
				(73–94.5%)	(60.8–76.8%)	(80.6% - 100%	(87% - 100%)	
	-ve	6	176					

+ve = positive, -ve = negative, CI = confidence interval.

Regarding agreement between saliva and NPS, the positive percent agreement(PPA), negative percent agreement (NPA) and overall percent agreement (OPA) between the two sampling methods were 83.8%, 92.6%, and 91.2% respectively with Kappa values of 0.7 (κ = 0.703, 95% CI 0.58–0.825). This, in turn, indicates substantial agreement between the two samples according to Kappa coefficient statistical interpretation cut-off value [22, 23]. In addition, the agreement between the two samples was statistically significant (*P* = 0.00)

### Evaluation of viral load concentration between saliva and NPS based on Ct-value

**Fig 1:**
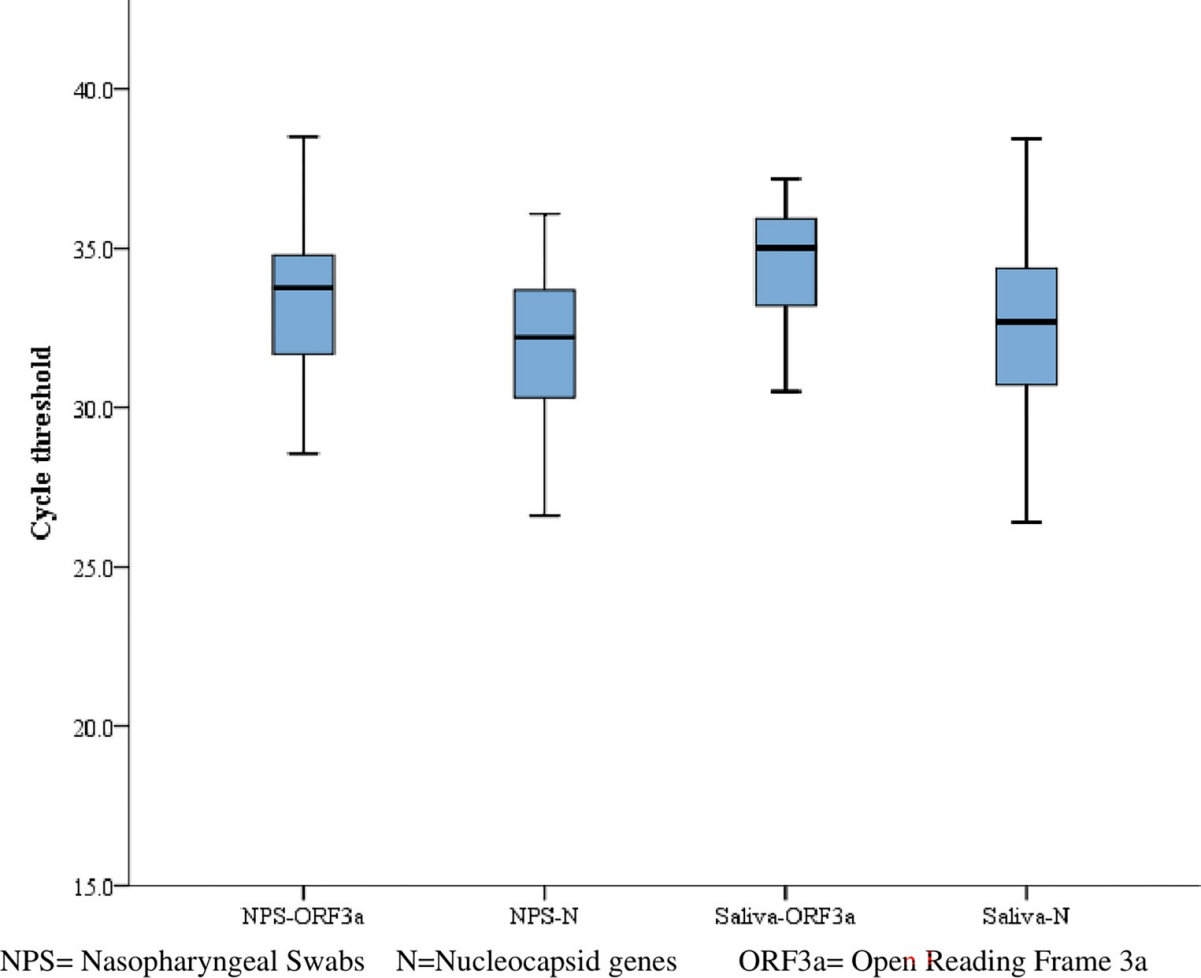
Cycle threshold values of paired ORF3a and N genes from NPS and Saliva samples for SARS-CoV-2 detection using RT-PCR among COVID-19 suspects.

The cycle threshold value was used as a proxy measurement for the viral load between the two samples whereby the viral load is inversely related. The mean Ct-values of ORF3a of NPS and saliva were 32.8 (SD±2.4) and 33.2 (SD±3.2) with mean difference of -0.36 (95% CI -1.7–0.98, P = 0.58), whereas Ct-values of paired N-genes of NPS and saliva were 31.7 (SD±2.9) and 31.1(SD±3.6) with mean difference of 0.64 (95%CI -0.71–1.99, P = 0.34) respectively. Moreover; the median Ct values of ORF3a and N genes for NPS were 33.75 (range; 28.55–38.50) and 32.19 (range; 26.60–36.07) respectively, whereas the median Ct-values of ORF3a and N genes for saliva were 35.00 (range; 23.81–38.18) and 32.68 (range; 22.29–38.43) respectively (r = 0.41, 95% CI -1.69–0.98, P >0.05) as shown in Fig 1. (**Fig 1)**

## Discussion

Real-Time qPCR remains the gold standard test for COVID-19 molecular diagnosis by detecting SARS-CoV-2 RNA and the NPS is the recommended sample [24–26]. However, our current finding showed higher detection and positivity rate in saliva for SARS-CoV-2 detection compared to NPS and this implies that saliva sample could serve as a good alternative for SARS-CoV-2 molecular diagnosis. This finding is supported by the recent announcement of US Food and Drug Administration toward approval of Saliva for SARS-CoV-2 RNA detection [27]. Saliva is an alternative specimen type that could eliminate the need for a swab and transport medium. It can potentially decrease the interaction time between healthcare providers and infected individuals, which could demand personal protective equipment usage and increase exposure [28].

In this study, the overall positivity rate of SARS-CoV-2 RNA in any sample among the study participants was 22.5% (51/227), (95% CI 17%– 28%) and this is in line with studies done in Switzerland (21.5%) [29] and the United States of America, (17.6%) [13]. However, much lower than the study conducted in Malaysia (73.7%) [30] and this difference might be due to variations in the study time. The current study was carried out in the late phase of the pandemic when there was declined COVID-19 incidence.

In addition, the separate positivity rate of SARS-CoV-2 RNA in saliva and NPS was 19.8% (45/227), (95% CI 14.5%– 25%), and 16.3% (37/227), (95% CI 11.5%– 21.1%) respectively. This is similar to the study conducted in Switzerland (19.9%, 20.87%) in saliva and NPS respectively [29]. Studies conducted in U.S.A [31] and Switzerland [32] had reported a positivity rate of 22.6% and 26.6% in saliva, 22.9% and 25.4% in NPS. Our finding is in line with these studies in terms of SARS-CoV-2 RNA positivity rate in saliva. However, the above-mentioned studies reported a higher positivity rate of SARS-CoV-2 in NPS than in saliva in contrast to our finding. This might be due to the difference in NPS collection skills since proper specimen collection is the most important step in the laboratory diagnosis of infectious diseases according to CDC guidelines. NPS collection is hindering and irritating, therefore, a wrong collection might lead to false negative results [11].

In our study, saliva showed higher sensitivity (83.8%, 95% CI, 73–94.5%) compared to NPS (68.8%, 95% CI 60.8–76.8%) with good PPA (83.8%), NPA (92.6%) and OPA of 91.2% with Kappa value of 0.7 (κ = 0.703, 95% CI 0.58–0.825). This is comparable with studies done in Addis Ababa, Ethiopia reported higher sensitivity in Saliva (92.14%) compared to NPS (52.63%) [33], Malaysia (93.1% vs 52.5%) [30], Singapore with a higher detection rate in saliva (62%) than in NPS (44.5%) [34], in Hong Kong (61.5% in saliva and 53.3% in NPS) [35] and Argentina where saliva showed higher sensitivity (98%) than NPS with Kappa value of 0.96 [36]. However, our study is uneven with the study conducted in the USA which reported lower sensitivity in saliva (91%) than NPS (98%) [37].

In addition, The sensitivity of saliva in our study (83.8%) is in line with systematic review and meta-analysis [38] and Wyllie and collegues [39], studies conducted in Saudi Arabia [40] and Japan [41] which had reported higher sensitivity in saliva than NPS. In our study, saliva showed comparable specificity (92.6%; 95% CI, 80.6% - 100%) with NPS (96.7%; 95% CI 87% - 100%). This finding is in line with a systematic review and meta-analysis conducted in Canada with pooled specificity of saliva and NPS 99.2% and 98.9% respectively [12], Brazil [42] and USA [43].

The overall agreement between saliva and NPS in our study (91.2%, κ *=* 0.703) is in agreement with the study done in Spain (93.3%, κ = 0.76) [44] but higher than the study conducted in Addis Ababa, Ethiopia 70% [33]. Our study reported a higher concordance rate (60.8%) than the study done in Malaysia (45.6%) [30]. This might be due to study participant differences as they included only asymptomatic study participants [30]. In our study, the concordance rate was in line with the study conducted by Ota and collegues with a concordance rate of 60.2% [45].

Based on viral load measurement, our study showed higher viral load in the NPS (median Ct-value of ORF3a and N genes was 33.75 and, 32.19 respectively) than in the saliva sample (median Ct-value of ORF3a and N gene 35.0 and 32.68 respectively). This is in line with studies conducted in the U.S.A [13], Australia [46] and Korea [47] which had reported higher viral load in NPS than in saliva samples. In contrast, our finding regarding the viral load between the two samples was not consistent with studies done in Malaysia [30] where there was a higher viral load in saliva than in NPS. In a study conducted in Addis Abba, Ethiopia [33], saliva showed a greater viral load than NPS. A study conducted in Thailand [48] reported a considerably similar viral load between saliva and NPS. In addition to this, a study conducted in Japan reported equivalent viral load between two samples in the earlier time but declined later in Saliva [3]. The potential reasons for these all differences might be due to differences in saliva collection methods, for instance, studies conducted in Addis Ababa, Ethiopia [33] and Malaysia [30] had collected early-morning saliva whereby patients were banned from washing their mouth, brushing their teeth, drinking and eating at all before saliva collection. In addition to this, the study coducted in Japan [3] collected saliva in two different rounds such as during the onset of the syndromes and at the convalescent phase after two weeks of the primary test.

SARS-CoV-2 genome encodes 29 proteins, 16 of which are nonstructural proteins (NSP1-NSP16), 4 of which are structural proteins (S, E, M, N) and nine of which are accessory ORFs (3a, 3b, 6, 7a, 7b, 8, 9b, 9c, and 10) [49]. ORF3a plays an important role in viral parthenogenesis and contributes to the severity of COVID-19 infection [50]. Furthermore, ORF3a codes for the largest proteins, which contain approximately 274 amino acids and it is also efficiently and effectively expressed on the cell surface. In this study, ORF3a gene was used to detect SARS-CoV-2 as it is easily detected in the majority of COVID-19 patients [51]. However, the E, RNA-dependent RNA polymerase (RdRp) and N genes were utilized for the detection of SARS-CoV-2 in the studies carried out in Switzerland [29], Malaysia [30] and Japan [41] respectively.

### Limitations of the study

The viral shedding time between the two samples was not assessed to differentiate how long saliva and NPS could still be positive for SARS-CoV-2. The NPS sample collection procedure might have caused the lower sensitivity observed in this current study since NPS collection is irritating and uncomfortable. The viral load of both saliva and NPS was measured only by Ct-value but copies/ml were not determined. Collecting deep-throat saliva from very young children (≤ 2 years) was tricky and the saliva obtained was more of by spitting method. In addition, NPS collection from young children was problematic too, therefore, unsuitable collection of the specimen might affect the sensitivity of the samples from these participants. The saliva collection was not restricted to timing, eating, and drinking for simplifying the collection. This might affect the detection rate in saliva for SARS-CoV-2.

## Conclusion and recommendation

Saliva showed greater sensitivity (83.8%) for SARS-CoV-2 RNA detection than NPS 68.8% though the difference was not statistically significant. There was a considerable concordance (60.8%) rate between NPS and saliva with higher overall percent agreement (92.6%, κ = 0.703). The agreement between the saliva and NPS samples was significant. Therefore, using saliva samples can still detect SARS-CoV-2 RNA; yielding a comparable result with NPS and could be used as an alternative specimen to NPS. As saliva can be collected by patients themselves, it could be an effective way to overcome the shortage of PPE and sample collection tools such as flocked swabs. Saliva could be considered as a diagnostic specimen for SARS-CoV-2 molecular diagnosis particularly where there is swab supply shortages and for children and patients who could barely give NPS. Further researchs evaluating viral shedding time and different saliva collection methods are recommended.

## Supporting information

S1 Data (XLSX)

## References

[1] Andersen K.G., et al., The proximal origin of SARS-CoV-2. Nature medicine, 2020. 26(4): p. 450–452. doi: 10.1038/s41591-020-0820-9 32284615 PMC7095063

[2] Lai C.-C., et al., Severe acute respiratory syndrome coronavirus 2 (SARS-CoV-2) and coronavirus disease-2019 (COVID-19): The epidemic and the challenges. International journal of antimicrobial agents, 2020. 55(3): p. 105924. doi: 10.1016/j.ijantimicag.2020.105924 32081636 PMC7127800

[3] Iwasaki S., et al., Comparison of SARS-CoV-2 detection in nasopharyngeal swab and saliva. Journal of Infection, 2020. 81(2): p. e145–e147. doi: 10.1016/j.jinf.2020.05.071 32504740 PMC7270800

[4] Rodriguez-Morales A.J., et al., History is repeating itself: Probable zoonotic spillover as the cause of the 2019 novel Coronavirus Epidemic. Infez Med, 2020. 28(1): p. 3–5. 32009128

[5] WHO. Coronavirus disease (COVID-19) pandemic. Situation report 2022 April,8,2022 [cited 2022 April 11, 2022]; Available from:https://www.who.int/emergencies/diseases/novel-coronavirus-2019?adgroupsurvey.

[6] Hanson K.E., et al., Infectious Diseases Society of America guidelines on the diagnosis of coronavirus disease 2019. Clinical infectious diseases, 2020. 10.1093/cid/ciaa760 PMC7337674 32556191

[7] Lee R.A., et al., Performance of saliva, oropharyngeal swabs, and nasal swabs for SARS-CoV-2 molecular detection: a systematic review and meta-analysis. Journal of clinical microbiology, 2021. 59(5). doi: 10.1128/JCM.02881-20 33504593 PMC8091856

[8] HCP H.P., Interim infection prevention and control recommendations for patients with suspected or confirmed coronavirus disease 2019 (COVID-19) in healthcare settings. National Center for Chronic Disease Prevention and Health Promotion. Division of Diabetes, Translation (Atlanta, GA, 2020)[Available from: https://stacks.cdc.gov/view/cdc/86043], 2020.

[9] Marty F.M., Chen K., and Verrill K.A., How to obtain a nasopharyngeal swab specimen. The New England journal of medicine, 2020. 382(22): p. e76–e76. doi: 10.1056/NEJMvcm2010260 32302471

[10] Vogels C.B., et al., SalivaDirect: Simple and sensitive molecular diagnostic test for SARS-CoV-2 surveillance. MedRxiv, 2020.

[11] CDC., C.f.D.C.a.P. Interim Guidelines for Collecting and Handling of Clinical Specimens for COVID-19 Testing. 2021 July, 2022 [cited 2022 5/8/2022]; Available from: https://www.cdc.gov/coronavirus/2019-ncov/lab/guidelines-clinical-specimens.html#print.

[12] Butler-Laporte G., et al., Comparison of saliva and nasopharyngeal swab nucleic acid amplification testing for detection of SARS-CoV-2: a systematic review and meta-analysis. JAMA internal medicine, 2021. 181(3): p. 353–360. doi: 10.1001/jamainternmed.2020.8876 33449069 PMC7811189

[13] Procop G.W., et al., A direct comparison of enhanced saliva to nasopharyngeal swab for the detection of SARS-CoV-2 in symptomatic patients. Journal of clinical microbiology, 2020. 58(11): p. e01946–20. doi: 10.1128/JCM.01946-20 32883744 PMC7587117

[14] To K.K.-W., et al., Temporal profiles of viral load in posterior oropharyngeal saliva samples and serum antibody responses during infection by SARS-CoV-2: an observational cohort study. The Lancet infectious diseases, 2020. 20(5): p. 565–574. doi: 10.1016/S1473-3099(20)30196-1 32213337 PMC7158907

[15] WHO., W.H.O. Laboratory testing for coronavirus disease 2019 (COVID-19) in suspected human cases. 2020 [cited 2022 7/7/2022]; Available from: https://apps.who.int/iris/bitstream/handle/10665/331329/WHO-COVID-19-laboratory-2020.4-eng.pdf.

[16] Berenger B.M., et al., Saliva collected in universal transport media is an effective, simple and high-volume amenable method to detect SARS-CoV-2. Clinical Microbiology and Infection, 2021. 27(4): p. 656–657. doi: 10.1016/j.cmi.2020.10.035 33160035 PMC7641592

[17] Leung E.C.m., et al., Deep throat saliva as an alternative diagnostic specimen type for the detection of SARS‐CoV‐2. Journal of medical virology, 2021. 93(1): p. 533–536. doi: 10.1002/jmv.26258 32621616 PMC7361401

[18] Misra V., et al., Guidelines for various laboratory sections in view of COVID-19: Recommendations from the Indian Association of Pathologists and Microbiologists. Indian Journal of Pathology and Microbiology, 2020. 63(3): p. 350. doi: 10.4103/IJPM.IJPM_857_20 32769321

[19] WHO., Laboratory testing for coronavirus disease 2019 (‎ COVID-19)‎ in suspected human cases: interim guidance, 2 March 2020. 2020, World Health Organization.

[20] Mico Biomed Co., L. Veri-Q nCoV-QM. COVID-19 multiplx detection kit 2020 [cited 2022 20/4/22]; Available from: http://www.micobiomed.com/en/index.php.

[21] Callahan C., et al., Saliva Is Comparable to Nasopharyngeal Swabs for Molecular Detection of SARS-CoV-2. medRxiv, 2021. doi: 10.1128/Spectrum.00162-21 34406838 PMC8552668

[22] Fleiss J.L., Levin B., and Paik M.C., Statistical methods for rates and proportions. 2013: john wiley & sons.

[23] Landis J.R. and Koch G.G., The measurement of observer agreement for categorical data. biometrics, 1977: p. 159–174. 843571

[24] Fan J., et al., Hock-a-loogie saliva as a diagnostic specimen for SARS-CoV-2 by a PCR-based assay: A diagnostic validity study. Clinica Chimica Acta, 2020. 511: p. 177–180. doi: 10.1016/j.cca.2020.10.004 33068630 PMC7557199

[25] Baron E.J., et al., A guide to utilization of the microbiology laboratory for diagnosis of infectious diseases: 2013 recommendations by the Infectious Diseases Society of America (IDSA) and the American Society for Microbiology (ASM) a. Clinical infectious diseases, 2013. 57(4): p. e22–e121. doi: 10.1093/cid/cit278 23845951 PMC3719886

[26] Ceron J.J., et al., Use of saliva for diagnosis and monitoring the SARS-CoV-2: a general perspective. Journal of clinical medicine, 2020. 9(5): p. 1491. doi: 10.3390/jcm9051491 32429101 PMC7290439

[27] Lisa W. First Saliva Test for COVID-19 Approved for Emergency Use by FDA|The Scientist Magazine®. 2020 [cited 2020 5/8/2022]; Available from: https://www.the-scientist.com/news-opinion/first-saliva-test-for-covid-19-approved-for-emergency-use-by-fda-67416.

[28] McCormick-Baw C., et al., Saliva as an alternate specimen source for detection of SARS-CoV-2 in symptomatic patients using Cepheid Xpert Xpress SARS-CoV-2. Journal of clinical microbiology, 2020. 58(8): p. e01109–20. doi: 10.1128/JCM.01109-20 32414838 PMC7383538

[29] Huber M., et al., High efficacy of saliva in detecting SARS-CoV-2 by RT-PCR in adults and children. Microorganisms, 2021. 9(3): p. 642. doi: 10.3390/microorganisms9030642 33808815 PMC8003663

[30] Rao M., et al., Comparing nasopharyngeal swab and early morning saliva for the identification of severe acute respiratory syndrome coronavirus 2 (SARS-CoV-2). Clinical Infectious Diseases, 2021. 72(9): p. e352–e356. doi: 10.1093/cid/ciaa1156 32761244 PMC7454325

[31] Landry M.L., Criscuolo J., and Peaper D.R., Challenges in use of saliva for detection of SARS CoV-2 RNA in symptomatic outpatients. Journal of Clinical Virology, 2020. 130: p. 104567. doi: 10.1016/j.jcv.2020.104567 32750665 PMC7392849

[32] Fougère Y., et al., Performance of RT-PCR on saliva specimens compared with nasopharyngeal swabs for the detection of SARS-CoV-2 in children: A prospective comparative clinical trial. The Pediatric infectious disease journal, 2021. 40(8): p. e300–e304. doi: 10.1097/INF.0000000000003198 34250969

[33] Beyene G.T., et al., Saliva is superior over nasopharyngeal swab for detecting SARS-CoV2 in COVID-19 patients. Scientific Reports, 2021. 11(1): p. 1–6. 34811429 10.1038/s41598-021-02097-2 PMC8608806

[34] Teo A.K.J., et al., Saliva is more sensitive than nasopharyngeal or nasal swabs for diagnosis of asymptomatic and mild COVID-19 infection. Scientific reports, 2021. 11(1): p. 1–8. 33542443 10.1038/s41598-021-82787-z PMC7862309

[35] Wong S.C.Y., et al., Posterior oropharyngeal saliva for the detection of severe acute respiratory syndrome coronavirus 2 (SARS-CoV-2). Clinical Infectious Diseases, 2020. 71(11): p. 2939–2946. doi: 10.1093/cid/ciaa797 32562544 PMC7337706

[36] Echavarria M., et al., Self‐collected saliva for SARS‐CoV‐2 detection: A prospective study in the emergency room. Journal of medical virology, 2021. 93(5): p. 3268–3272. doi: 10.1002/jmv.26839 33527375 PMC8013278

[37] Czumbel L.M., et al., Saliva as a candidate for COVID-19 diagnostic testing: a meta-analysis. Frontiers in medicine, 2020. 7: p. 465. doi: 10.3389/fmed.2020.00465 32903849 PMC7438940

[38] Lee R.A., et al., Performance of saliva, oropharyngeal swabs, and nasal swabs for SARS-CoV-2 molecular detection: a systematic review and meta-analysis. Journal of clinical microbiology, 2021. 59(5): p. e02881–20. doi: 10.1128/JCM.02881-20 33504593 PMC8091856

[39] Wyllie A.L., et al., Saliva is more sensitive for SARS-CoV-2 detection in COVID-19 patients than nasopharyngeal swabs. MedRxiv, 2020.

[40] Khurshid Z., Asiri F.Y.I., and Al Wadaani H., Human saliva: non-invasive fluid for detecting novel coronavirus (2019-nCoV). International journal of environmental research and public health, 2020. 17(7): p. 2225. doi: 10.3390/ijerph17072225 32224986 PMC7178089

[41] Sakanashi D., et al., Comparative evaluation of nasopharyngeal swab and saliva specimens for the molecular detection of SARS-CoV-2 RNA in Japanese patients with COVID-19. Journal of Infection and Chemotherapy, 2021. 27(1): p. 126–129. doi: 10.1016/j.jiac.2020.09.027 33060046 PMC7524660

[42] Vaz S.N., et al., Saliva is a reliable, non-invasive specimen for SARS-CoV-2 detection. Brazilian Journal of Infectious Diseases, 2020. 24: p. 422–427. doi: 10.1016/j.bjid.2020.08.001 32888905 PMC7458056

[43] Warsi I., et al., Saliva exhibits high sensitivity and specificity for the detection of SARS-COV-2. Diseases, 2021. 9(2): p. 38. doi: 10.3390/diseases9020038 34065171 PMC8161819

[44] Fernández-González M., et al., Performance of Saliva Specimens for the Molecular Detection of SARS-CoV-2 in the Community Setting: Does Sample Collection Method Matter? Journal of clinical microbiology, 2021. 59(4): p. e03033–20. doi: 10.1128/JCM.03033-20 33419948 PMC8092755

[45] Ota K., et al., Detection of SARS-CoV-2 using qRT-PCR in saliva obtained from asymptomatic or mild COVID-19 patients, comparative analysis with matched nasopharyngeal samples. PLoS One, 2021. 16(6): p. e0252964. doi: 10.1371/journal.pone.0252964 34111203 PMC8191987

[46] Williams E., et al., Saliva as a noninvasive specimen for detection of SARS-CoV-2. Journal of clinical microbiology, 2020. 58(8): p. e00776–20. doi: 10.1128/JCM.00776-20 32317257 PMC7383524

[47] Yoon J.G., et al., Clinical significance of a high SARS-CoV-2 viral load in the saliva. Journal of Korean medical science, 2020. 35(20). doi: 10.3346/jkms.2020.35.e195 32449329 PMC7246183

[48] Pasomsub E., et al., Saliva sample as a non-invasive specimen for the diagnosis of coronavirus disease 2019: a cross-sectional study. Clinical Microbiology and Infection, 2021. 27(2): p. 285. e1–285. e4. doi: 10.1016/j.cmi.2020.05.001 32422408 PMC7227531

[49] Zhang J., et al., Understanding the role of SARS-CoV-2 ORF3a in viral pathogenesis and COVID-19. Frontiers in microbiology, 2022. 13: p. 854567. doi: 10.3389/fmicb.2022.854567 35356515 PMC8959714

[50] Bianchi M., et al., SARS-CoV-2 ORF3a: mutability and function. International journal of biological macromolecules, 2021. 170: p. 820–826. doi: 10.1016/j.ijbiomac.2020.12.142 33359807 PMC7836370

[51] Tsai P.-H., et al., Genomic variance of Open Reading Frames (ORFs) and Spike protein in severe acute respiratory syndrome coronavirus 2 (SARS-CoV-2). Journal of the Chinese Medical Association, 2020. 83(8): p. 725. doi: 10.1097/JCMA.0000000000000387 32773643 PMC7493783

